# Phylogeographic variation in recombination rates within a global clone of methicillin-resistant *Staphylococcus aureus*

**DOI:** 10.1186/gb-2012-13-12-r126

**Published:** 2012-12-27

**Authors:** Santiago Castillo-Ramírez, Jukka Corander, Pekka Marttinen, Mona Aldeljawi, William P Hanage, Henrik Westh, Kit Boye, Zeynep Gulay, Stephen D Bentley, Julian Parkhill, Matthew T Holden, Edward J Feil

**Affiliations:** 1 Department of Biology and Biochemistry, University of Bath, Claverton Down Bath, Bath and North East Somerset BA2 7AY, UK; 2 Department of Mathematics and Statistics, PO Box 68 (Gustaf Hällströmin katu 2b), University of Helsinki, FI-00014 Helsinki, Finland; 3 Department of Information and Computer Science, Helsinki Institute for Information Technology HIIT, Aalto University, PO Box 15400 (Konemiehentie 2), FI-00076 Aalto, Finland; 4 Department of Biomedical Engineering and Computational Science, Aalto University, PO Box 12200 (Rakentajanaukio 2c), FI-00076 Aalto, Finland; 5 Center for Communicable Disease Dynamics, Department of Epidemiology, Harvard School of Public Health, 677 Huntington Avenue, Boston, MA 02115, USA; 6 Department of Clinical Microbiology 445, Hvidovre Hospital, DK-2650 Hvidovre, Denmark; 7 Faculty of Health Sciences, University of Copenhagen, Blegdamsvej 3B, DK-2200 Copenhagen, Denmark; 8 Dokuz Eylul University School of Medicine, Department of Clinical Microbiology, Mithatpaşa cad., Inciralti, Izmir 35340, Turkey; 9 Wellcome Trust Sanger Institute, Wellcome Trust Genome Campus, Hinxton, Cambridgeshire CB10 1SA, UK

## Abstract

**Background:**

Next-generation sequencing (NGS) is a powerful tool for understanding both patterns of descent over time and space (phylogeography) and the molecular processes underpinning genome divergence in pathogenic bacteria. Here, we describe a synthesis between these perspectives by employing a recently developed Bayesian approach, BRATNextGen, for detecting recombination on an expanded NGS dataset of the globally disseminated methicillin-resistant *Staphylococcus aureus *(MRSA) clone ST239.

**Results:**

The data confirm strong geographical clustering at continental, national and city scales and demonstrate that the rate of recombination varies significantly between phylogeographic sub-groups representing independent introductions from Europe. These differences are most striking when mobile non-core genes are included, but remain apparent even when only considering the stable core genome. The monophyletic ST239 sub-group corresponding to isolates from South America shows heightened recombination, the sub-group predominantly from Asia shows an intermediate level, and a very low level of recombination is noted in a third sub-group representing a large collection from Turkey.

**Conclusions:**

We show that the rapid global dissemination of a single pathogenic bacterial clone results in local variation in measured recombination rates. Possible explanatory variables include the size and time since emergence of each defined sub-population (as determined by the sampling frame), variation in transmission dynamics due to host movement, and changes in the bacterial genome affecting the propensity for recombination.

## Background

The wave of molecular data generated through the advent of full genome sequencing and molecular typing methodologies such as multilocus sequence typing (MLST) has revolutionized our understanding of micro-evolutionary dynamics in bacterial pathogens. Although MLST has proved very successful both as a typing scheme for epidemiological surveillance and for elucidating population structure, these data are based on <1% of the genome and thus have limited resolving power for elucidating patterns of dissemination and microevolution within very recently emerged clones. However, the recent deployment of next-generation sequencing (NGS) platforms for phylogeographic studies has provided the means to generate ultra-high resolution SNP data (covering >95% of the genome) for large population samples. This technology has shed light on the divergence of highly virulent or resistant clones over years or months [1-3] within a defined locale [4,5], transmission within a single health-care setting [6,7] and even the molecular changes associated with prolonged carriage and invasive disease in a single host [8]. Such studies have laid the foundation for a new era for evolutionary biology and molecular epidemiology in bacterial pathogens, particularly when considered alongside extensive metadata and social contact networks [9].

In addition to understanding long- and short-term transmission dynamics, NGS data can also inform on questions relating to molecular processes such as mutation and recombination, and the evolutionary fate of this variation [10]. Current studies tend to emphasize one of these perspectives over the other. For example, Croucher *et al. *[1] comprehensively described the role and clinical significance of recombination within a resistant clone of *Streptococcus pneumoniae*, but did not explicitly consider the epidemiological context of the isolates. In contrast, the study of Harris *et al. *[2] in the hospital-acquired methicillin-resistant *Staphylococcus aureus *(MRSA) clone ST239 provided detailed evidence concerning inter-continental transmission, but had less focus on recombination. However, just as horizontal gene transfer may impact on the epidemiological properties of a clone (for example, via the acquisition of a gene conferring heightened virulence [11]), so the dynamics of clonal dissemination, due to factors such as host movement, will in turn impact on genome evolution. For example, the conditions necessary for sub-populations to first diversify, and then subsequently recombine, will clearly be determined by the rates of transmission over a range of geographical scales.

Here we describe a novel synthesis between global patterns of dissemination and diversification (phylogeography) and the relative rates of recombination (compared to mutation) within the hospital-acquired MRSA clone ST239. We supplement the data of Harris *et al. *with a further 102 isolates, and employ a recently developed Bayesian method for identifying recombination events on a genome-wide scale, both within the stable core and in the non-core (accessory) genome. Thirty-one of the extra isolates are from a global collection and 71 were recovered from 4 Turkish hospitals, thus providing detailed evidence as to the transmission dynamics within this country. We considered Turkey to be an excellent focal country for this study as it has been described as the 'epicenter' of ST239, owing to the extreme predominance of this clone (>95%) amongst hospital-acquired MRSA infections [12]. We also considered that the location of Turkey straddling the border of Europe and Asia would provide evidence as to the spread of this clone out of Eastern Europe. Our analysis indicates significant variation in the relative impact of recombination (compared to mutation) between the ST239 sub-groups associated with different geographical regions.

## Results

### Patterns of diversity and recombination in the core and non-core genomes

We supplemented the original global data of Harris *et al. *(62 isolates) [2] with full-genome SNP data from a further 31 isolates from a global collection and 71 isolates from Turkey (Additional file 1). The Turkish isolates were recovered from four hospitals representing the three largest cities in Turkey; Istanbul, Ankara and Izmir. The combined dataset of 165 isolates (62 previously sequenced + 102 newly sequenced + the reference genome, *S. aureus *TW20, [13]) resulted in a total of 16,172 high-quality SNPs. These SNPs were derived both from the previously defined core regions [2], which are present in all strains, but also from the non-core regions [2], which are variably present or absent, and correspond largely to mobile genetic elements (MGEs). Although recombination is known to occasionally affect the core genome of *S. aureus *[14,15], this occurs at a low rate compared to other bacterial pathogens [16]. In contrast, non-core genes are highly mosaic and contain a much higher SNP density than core genes, indicative of high rates of recombination [2,10].

**Figure 1:**
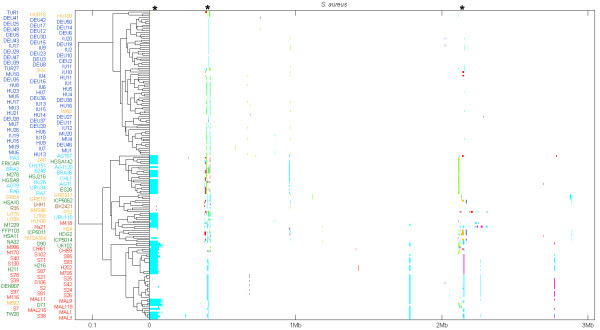
**Results of the recombination analysis for the 165 ST239 isolates**. The names of the strains are shown on the left and are colored according to the geographic source as follows: red, Asia; yellow, Eastern Europe; green, Western Europe; cyan (light blue), South America; dark blue, Turkey. The colored bars in the panel on the right side of the dendrogram denote the recombination events in the strains along the genome. The coloring of the bars at a specific genomic location reflects the clustering of the recombination events into groups, and is unrelated to either the coloring of the strain names or other bars at distant genomic locations. The asterisks indicate well-known MGEs: the SCCmec element, the phage φSa1 (TW20) and the phages φSaβ-TW20/φSpβ-like.

We used the Bayesian Recombination Tracker (BRATNextGen) program [17] to detect recombination events within the 165 isolates. Details of all the recombination events identified are given in Additional file 2, and a graphical summary is shown in Figure 1. Of the 16,172 SNPs (corresponding to 0.53% of the genome) identified, 8,569 (53%) have been introduced by recombination. Additional evidence for recombination within the genome as a whole was also generated using the phi test and by constructing a neighbor-net, as implemented in SplitsTree4 [18] (Additional file 3). As expected, the vast majority of recombination events have affected non-core MGEs. Figure 1 illustrates that the recombination events are non-randomly distributed throughout the genome, and three strikingly recombinogenic regions are evident (indicated by asterisks). These regions correspond to well-known non-core MGEs; the SCCmec element (approximately 30 kb from the origin), the phage φSa1 (TW20) (approximately 450 kb from the origin) and the phages φSaβ-TW20/φSpβ-like at approximately 2.2 Mb.

**Figure 2:**
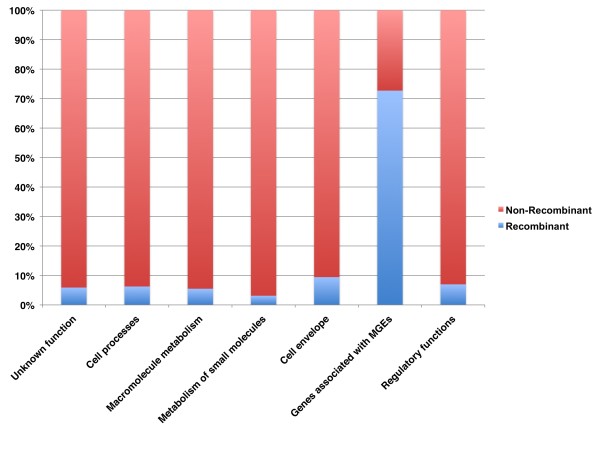
**Functions affected by recombination**. The bars are the most inclusive categories based on an adapted version of Riley's classification. The blue section of the bars represents the percentage of genes of each functional category affected by recombination. The 'extrachromosomal' category was here renamed 'MGE' to avoid confusion as these genes are physically located on the chromosome.

We next identified all those genes that have been affected by recombination on the basis they contain at least 100 bp that are also located on a recombinant segment as defined by the BRATNextGen analysis. A total of 441 (15.3%) of the 2,869 genes in the reference genome (TW20) met this criterion (Additional file 4) and the proportion of genes affected by recombination within different Riley's functional categories [19] was examined. When MGEs are excluded, the percentage of genes affected by recombination ranges from approximately 3% to approximately 10%, with the highest rate evident in those genes encoding proteins associated with the cell envelope (Figure 2). In contrast, over 70% of genes associated with MGEs have been affected by recombination. We then considered the ratio of nucleotide changes as a result of recombination relative to point mutation (r/m ratio) for the MGEs and core by assigning SNPs as having arisen through recombination if they reside on a recombinant segment according to BRATNextGen, and through mutation if not. This approach provides a measure of the contribution of recombination and mutation to genome diversification. Recombination events between identical sequences are not counted, as they are impossible to detect and do not introduce any variation. Rather than use Riley's gene functional categories, MGEs in this analysis (as well as all those hereafter) were defined directly on the annotated features of the TW20 genome [13]; the genomic coordinates of genes assigned as MGEs are given in Additional file 5. When all mapped genes were considered, we calculated an r/m ratio of 1.13 from the BRATNextGen output, which decreased to 0.11 when the MGEs were excluded. We note that this latter estimate is consistent with previous estimates based on MLST (core) data [14]. Although our analysis confirms a far higher rate of recombination in the non-core than in the core, it is not possible to draw robust comparisons between the two as our analysis is based on mapping to a single reference genome and some of the variation present in the MGEs will be excluded.

### The global phylogeography of ST239

The identification of recombination events also facilitates robust phylogenetic analysis through their subsequent exclusion. Although the impact of recombination on the core genome was confirmed to be low using BRATNextGen, we supplemented this analysis with a second method for detecting recombination recently applied to NGS data of *S. pneumoniae *[1]. We took a conservative approach and excluded SNPs corresponding to recombined regions in either one, or both, of these approaches for use in downstream phylogeographic analyses. The number of genes that were found to be affected by recombination in both analyses was 312 (compared with 441 on the basis of BRATNextGen alone; Additional file 6), but we note that the distribution of these genes according to functional category was similar as described previously, with the vast majority of recombined genes corresponding to MGEs, and higher proportion of cell envelope genes than other core categories (Additional file 7).

**Figure 3:**
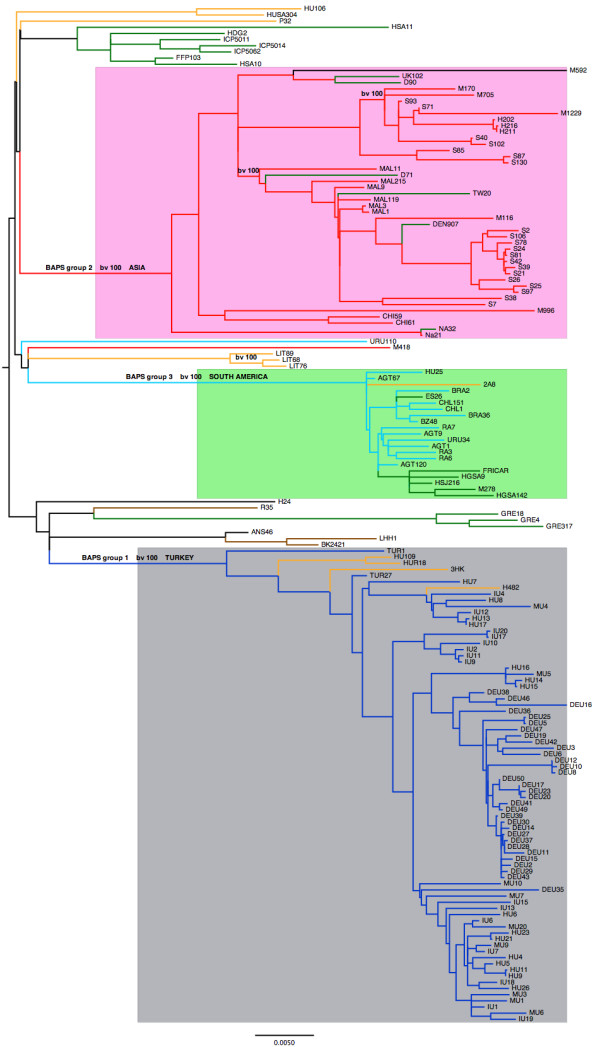
**The maximum likelihood phylogeny of the ST239 isolates**. The tree is based on the SNPs that were not affected by recombination using either BRATNextGen or the method used in [1]. The color coding of the branches is as follows: red, Asia; yellow, Eastern Europe; green, Western Europe; cyan (light blue), South America; dark blue, Turkey; brown, USA; the black branches refer to Australia (ANS46), Egypt (H24), and Syria (M592). The particular country of origin for each isolate is given in Additional file 1. The grey rectangle shows all the strains within Bayesian Analysis of Population Structure (BAPS) group number one (Turkish clade), the pink rectangle covers the strains composing BAPS group number 3 (Asian clade), and the light green rectangle shows the strains found in BAPS group number 2 (South American clade). For the sake of clarity the bootstrap values (bv) are only shown for the three main clades and some minor groups. The scale bar represents substitutions per SNP.

Figure 3 shows a maximum likelihood phylogenetic analysis of the isolates listed in Additional file 1. This phylogeny is based on the SNPs that were not affected by recombination. The tree is color coded according to geographical region, and the alignment used to construct the tree is given in Additional file 8. Discounting the Turkish isolates, the extra 31 global isolates used in this analysis confirms the robustness of two major monophyletic clades, which are supported by 100% bootstrap scores. Although these two clades correspond mainly to Asian and South American isolates, there are clear discrepancies such as the cluster of European origin within the South American clade. As noted previously [2], many European isolates form more diverse, basal groups consistent with a European origin, but this expanded dataset also reveals further details concerning the phylogeography of this clone as discussed below.

Harris *et al. *noted that 20 isolates from a single hospital in northern Thailand resolved into two distinct clades. The current data reveal that isolates from Malaysia (MAL 1, 3, 119, 9, 215, 11) cluster basally, with 100% bootstrap support, to one of these clades but not the other. This indicates that this clade may have been introduced into Thailand via Malaysia (or another neighboring country). Additional Thai isolates (M170, M705, H202) cluster with the second Thai clade, suggesting this clade represents an older and more established population of ST239 in Thailand. Whereas the study of Harris *et al. *focused on two extremes of geographical scale (global and a single hospital), the current data also reveal evidence for strong structuring on an intermediate, countrywide, scale. For example, three isolates from Lithuania (LIT 89,76,68) form a tight cluster, having a bootstrap value of 100, which is distinct from other European isolates, and three newly sequenced isolates from Argentina (RA3, 6, 7) cluster with four original isolates from this country (AGT 120, 9, 1). Although in this case the bootstrap value (44) is low, this is consistent with a distinct Argentinean sub-cluster within the South American group.

Most striking, however, is the observation that the Turkish isolates form a single monophyletic cluster. There are no Turkish isolates that fall outside this cluster. The two isolates of Turkish origin from the original study of Harris *et al. *(TUR 1, 27) are positioned basally within this cluster, which is expected as they were isolated a decade earlier (1995/1996) than the newly characterized isolates (2006/2007). Four isolates of Eastern European origin are also clustered basally within this group (yellow branches within the Turkish clade; HU109, 7, HUR18 (Hungary), HK3 (Czech Republic), H482 (Romania)). Taken together, these observations provide strong evidence of a single introduction of ST239 into Turkey from Eastern Europe, with very limited transmission of ST239 into or out of Turkey subsequent to this event.

### The phylogeography of ST239 within Turkey

**Figure 4:**
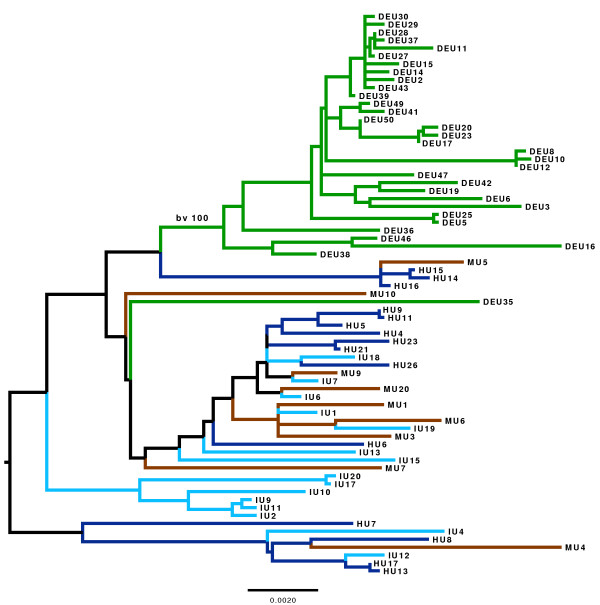
**The maximum likelihood tree of the newly sequenced Turkish isolates**. Branch color coding is as follows: green, Izmir; dark blue, Ankara; light blue, Istanbul European side; brown, Istanbul Asian side. Only the 71 newly sequenced isolates from Turkey were considered and so the Turkish isolates used in the study by Harris *et al. *[2] and the western European isolates were excluded for this analysis. The scale bar represents substitutions per SNP. Only for the Izmir group the bootstrap value (bv) is given for simplicity's sake.

To further study the geographical structuring on a national scale, we constructed another phylogeny based on those SNPs not affected by recombination and only using the 71 newly sequenced Turkish isolates that were isolated from 4 major university hospitals. Two of these were in Istanbul, one on the European side of the Bosphorus (Istanbul University Cerrahpasa Hospital, *n *= 15; IU) and the other on the Asian side (Marmara Hospital, *n *= 9; MU). The other two sampling locations were Hacettepe University Hospital in Ankara (*n *= 15; HU), and Dokuz Eylul University hospital in Izmir (*n *= 32; DEU). Figure 4 presents the phylogenetic tree color coded according to hospital. Whereas the isolates from the three hospitals in Istanbul and Ankara are intermingled on the tree, all but one (DEU 35) of the isolates from Izmir form a distinct cluster with 100% bootstrap support (green branches), and no isolates from the Ankara or Istanbul hospitals are positioned within this cluster. This suggests frequent transmission of MRSA between hospitals in Ankara and Istanbul, but a single founder event into the hospital in Izmir and very limited subsequent transmission to and from other hospitals in Turkey. These inferences are consistent with patterns of host (human) movement between these cities; Ankara and Istanbul are well connected whereas Izmir is relatively isolated. In support of this argument, we note that the total number of domestic flights from Izmir to Istanbul and Ankara was 259 and 262, respectively, during the week 2 to 8 Nov 2012 (Additional file 12), whereas the total number of flights between Istanbul and Ankara in the same period was 450.

### Dating the population sub-groups

In order to further define the global structure of the ST239 population, we used the program Bayesian Analysis of Population Structure (BAPS) [20]. This approach resolved the three major groups revealed by the phylogenetic analysis, corresponding to the Turkish clade (including the eastern European isolates), South American, Asian and a fourth group that mainly contains isolates from Europe and USA (the strains composing each group are listed in Additional file 9). Of these, only the fourth group is polyphyletic; the three BAPS groups that are monophyletic are highlighted by the colored rectangles in Figure 3. We used the Bayesian Evolutionary Analysis by Sampling Trees (BEAST) program [21] to construct a calibrated phylogeny (Additional file 10), this phylogeny suggests that ST239 first emerged in Europe in 1965 (late 1960, early 1973), which is consistent with the previous analysis by Harris *et al. *[2]. The observation that the three monophyletic clades appear unrelated to each other points to multiple independent exports from Europe, rather than a progressive wave of global dissemination. We estimate that the Turkish and Asian clades both emerged around 1982 (Turkish clade 1982 (1975, 1988) and Asian clade 1983 (late 1976, 1989)), whereas we estimate the introduction into South America to have occurred approximately a decade later in 1992 (late 1989, 1993) (Additional file 10).

### Recombination between and within groups

Given the phylogeographic framework described above, we then examined the impact of recombination between the four BAPS sub-groups, and separately within each sub-group. An admixture analysis revealed very little evidence for recombination between the groups (Table 1). Only 10 out of the 165 isolates show significant evidence of admixture. These include two pairs of closely related isolates and a cluster that comprises the three isolates from Lithuania, which are likely to represent single events; thus, a minimum of six recombination events have been detected. We note that isolates from the fourth group appear to show the greatest admixture, and in particular appear to have imported DNA from Asian isolates.

**Table 1:** Admixture analysis between groups

Isolate	Turkish (BAPS group 1)	Asian (BAPS group 2)	South American (BAPS group 3)	Fourth (BAPS group 4)	*P*-value
ANS46	0.25	0	0	0.75	0
CHI61^a^	0	0.96	0.04	0	0.01
CHI59^a^	0	0.77	0.19	0.04	0
India	0	0.2	0	0.8	0.005
LIT68^b^	0	0.17	0.05	0.78	0
LIT76^b^	0	0.18	0.03	0.79	0
LIT89^b^	0	0.18	0.08	0.74	0
NA32^c^	0	0.41	0	0.59	0
Sri Lanka^c^	0	0.22	0	0.78	0
TUR1	0.76	0	0.03	0.21	0

Isolates showing significant evidence for admixture. Columns 2 to 5 show the Bayesian posterior mean estimates of the proportion of SNPs represented by the BAPS groups. Only *P*-values <0.05 were considered significant and only those cases are shown. ^a^Cluster together in the ML phylogeny and therefore might be just one event. ^b^Cluster together in the ML phylogeny and therefore might be just one event. ^c^Cluster together in the ML phylogeny and therefore might be just one event.

**Figure 5:**
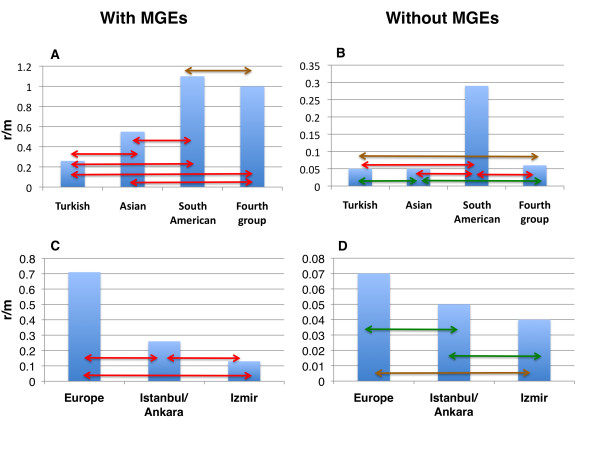
**The r/m ratios for the BAPS groups and for the analysis of two founder events**. **(a,b)** The r/m figure for each BAPS group with (a) and without MGE (b). (c,d) The r/m values for the two founder events within the Turkish group with (c) and without MGE (d). The arrows specify the comparisons between the different sets. Red arrows show comparisons that are significant at <0.0001, brown arrows those significant at <0.05 and green arrows those that are not significant (P-value >0.05). Of note, the scale of the y-axis is different for each panel.

For each BAPS sub-group we calculate two r/m ratios, one including the major MGEs and one with the MGEs excluded (Table 2). We used a chi-squared test to compare the number of SNPs introduced by recombination and mutation between each pair of sub-populations (Figure 5a,b). When the MGEs are included (Figure 5a), all pairwise comparisons between the four different sub-groups (fourth, Asian, Turkish and South American) show highly significant differences (*P *< 0.0001; red arrows) except for the fourth sub-group versus South American (*P *< 0.05; brown arrow). The fourth sub-group and South American show the highest r/m, Turkish the lowest, and Asian intermediate. When the MGEs are excluded (Figure 5b), r/m drops dramatically for the fourth, Asian and Turkish sub-groups but remains higher for the South American sub-group (*P *< 0.0001 against the other three groups; red arrows). The r/m for the fourth sub-group remains significantly higher than for the Turkish sub-group (*P *= 0.015; brown arrow), with the Asian sub-group again in an intermediate position (but not significantly different from either the fourth sub-group or the Turkish sub-group; green arrows). This analysis thus indicates a significant difference in recombination rates (relative to mutation) between the South American sub-group and the other sub-groups, and broadly consistent trends between the other sub-groups, regardless of whether the MGEs are included. We then considered the impact on r/m of two founder events inferred from the data; first, the introduction of ST239 into Turkey from Eastern Europe and, second, the subsequent dissemination within Turkey to Izmir on the west coast. Figure 5c,d show the r/m estimates for three subpopulations (Additional file 11): Europe (EUR), Istanbul/Ankara (I/A), and Izmir (IZ). We removed a single European isolate (P32 from Poland) from this analysis as this exhibits an elevated mutation rate consistent with hypermutation. Our estimates of r/m based on the BRATNextGen output indicate a decrease in r/m over both founder events, first from EUR to I/A and second from I/A into IZ. This trend is observed when the MGEs are included or excluded. Although the differences between EUR and I/A, and I/A and IZ are only significant when the MGEs are included (Figure 5c; *P *< 0.0001), there remains a significant difference between EUR and IZ on the core data alone (Figure 5d; *P *= 0.023; brown arrow). This analysis is consistent with lowered rates of recombination or, equivalently, elevated rates of mutation within very recently emerged populations. We note that it is impossible to detect recombination events between identical sequences, meaning that the true rate of recombination is underestimated. However, the contribution of recombination and mutation to clonal diversification is captured by r/m.

**Table 2:** The r/m ratios

Group	r/m with MGE	r/m without MGE
South America	1.1 (m 1839, r 2032)	0.29 (m 895, r 264)
Asia	0.55 (m 3080, r 1707)	0.05 (m 2071, r 104)
Turkey	0.26 (m 3207, r 839)	0.05 (m 2017, r 92)
Fourth group	1 (m 4276, r 4277)	0.06 (m 2730, r 173)

For each BAPS group the r/m ratio was calculated with and without MGE. m: SNPs introduced by mutation; r: SNPs introduced by recombination

## Discussion

The principal goal of this study was to demonstrate the utility of NGS data for simultaneously exploring both molecular processes and phylogeographic patterns in pathogenic bacteria over very short time scales. Our data confirm that the overwhelming majority of recombination is restricted to the highly diverse non-core genes corresponding to MGEs and points to similar rates of recombination in the core genome suggested previously using MLST data [14,16,22]. Somewhat higher estimates have recently been published from full genomic comparisons [23,24], but this discrepancy might be due to the inclusion of non-core genes in these studies, or other methodological differences. For example, we note that in the study by Chan *et al. *[23], hypothetical proteins and genes of unknown function categories were overrepresented in whole-gene transfers. Our data also point to higher rates of recombination in core genes encoding proteins associated with the cell envelope than in other core categories, which is consistent with the view that such genes tend to be under higher levels of diversifying selection [25]. We note that the analysis of the non-core is limited by read-mapping to only a single ST239 reference genome (TW20). This means that any MGEs present in other ST239 genomes, but absent in TW20, are not taken into account, and the true diversity of the non-core is under-estimated. Nevertheless, the comparisons we draw between the core and non-core remain indicative of general trends between sub-groups, as discussed below. We also do not consider the lack of alternative reference genomes to be a problem for capturing core genome divergence. We note that the very recent emergence of ST239 means that the ST239-specific core will represent a higher proportion of the whole genome than the core of the species as a whole. Thus, broadly speaking, we consider a single reference genome to be sufficient for a detailed analysis of core divergence on this very fine micro-evolutionary scale, and for generating useful, if incomplete, evidence concerning the non-core.

In order to objectively define population sub-groups prior to comparative analysis, we used a combination of robust phylogenetic reconstruction using maximum likelihood, and a Bayesian approach for defining sub-groups implemented in BAPS. As discussed previously [20], this integrated approach is critical. Without the use of BAPS it would be unclear as to which clusters on the tree could be objectively ring-fenced and promoted to a sub-group, and without the phylogenetic analysis it would not be clear which of the BAPS sub-groups are monophyletic. The four sub-groups defined by BAPS in our analysis show strong geographical correspondence, being composed primarily of isolates from Asia, South America, Turkey, and a fourth sub-group consisting mainly of strains from Europe and USA. Three of these sub-groups are robustly supported by the phylogeny; the fourth BAPS group (Europe + USA) is an exception as it is polyphyletic and is defined on the basis that the isolates do not belong in any of the other groups. Without the accompanying phylogeny, the nature of this fourth sub-group would not be obvious.

The power of any population level analysis is ultimately dependent on the sampling frame, and we note that the promotion of a phylogenetic cluster to a population sub-group, through the use of BAPS, is largely dependent on how well the cluster is represented in the sample. For example, the three isolates from Lithuania form a clear monophyletic cluster but, unlike the Turkish cluster, they are not defined as a population sub-group by BAPS, presumably because there are so few isolates. To examine this more quantitatively, we re-ran the BAPS analysis excluding random isolates from the Turkish cluster, in order to gauge the approximate number of Turkish isolates required for this cluster to be defined as a BAPS sub-group. When the analysis was run with only 20 or 15 Turkish isolates, the same four BAPS groups were recovered, but when only 5 or 10 Turkish isolates were included, just three groups were defined and the Turkish isolates were assimilated into the fourth group. This suggests that, at least for our dataset, between 10 and 15 isolates are required before a monophyletic cluster is assigned as a population sub-group by BAPS, even if it is a cluster defined by a long branch.

We then combined the two strands of analysis by considering the relative impact of recombination compared to mutation (r/m) in the core and non-core within different population sub-groups. This analysis demonstrated statistically significant variation in r/m between the population sub-groups, thus highlighting an association between molecular processes and phylogeography. Although these differences were far more striking when MGEs were considered, our data suggest broadly similar trends when these elements are excluded. Gene flow has previously been shown to be structured in bacterial populations [26,27], and links have been drawn with shifts in epidemiological properties. For example, epidemic serogroup A *Neisseria meningitidis*, which has caused pandemic waves in sub-Saharan Africa [28], appears to be recombining at a lower rate than other serogroups, and it has been proposed that this may be due to the rapid spread of this clone [29]. However, to our knowledge the current study represents the first attempt to consider how recombination varies within a single clone (which has emerged and globally disseminated in under 50 years), and within the context of a robust phylogeographic framework. Below, we discuss three broad (and non-exclusive) classes of explanation for this phylogeographical variation in recombination: time-dependence, variation in transmission associated with host movement, and changes in the bacterial genome.

Regarding the possibility of time-dependence, we consider if there are any *a priori *expectations that a very recently emerged local population should show lower rates of recombination (relative to mutation) than a more established one. A nascent population expanding from a single founder event will initially contain very low levels of diversity. If recombination mostly occurs within the clone, the statistical detection of recombination events within a sample drawn from a very recently emerged population will prove problematic, and detectable levels of recombination should increase over time as diversity accumulates in the population. This then might explain the decrease in r/m associated with the founder events from Eastern Europe into Turkey and within Turkey to Izmir (Figure 5c,d). An important caveat is that this argument assumes very limited recombination between ST239 and other *S. aureus *lineages carried asymptomatically on the skin or in the anterior nares. ST239 is multiply antibiotic resistant, and generally very rarely observed outside of the hospital environment (with the notable exception of a recently described variant in Russia [30]). Further, at least 90% of the hospital-acquired MRSA within Turkey correspond to this clone [12]; thus, this ecological specialism of ST239 may limit the opportunities for recombination with other lineages. Genetic factors such as sequence divergence and the presence of lineage-specific restriction modification systems have also been proposed to impact on the relative rates of recombination within and between lineages [31,32]. On the other hand, it is also clear that recombination between different *S. aureus *lineages does occur occasionally, the hybrid genome of ST239 being a striking example [15].

It is also interesting to speculate as to whether differences in the selective consequences of recombination and *de novo *mutation might be playing a role, as this might also result in a time-dependent effect. For example, if *de novo *mutations were, on average, more selectively deleterious than recombination events, then these would be preferentially purged over time and the r/m would increase. In support of this, we have recently shown that the variation imported by recombination is largely synonymous [10], hence possibly less likely to be deleterious than a *de novo *non-synonymous mutation that has not passed through a selective filter.

Arguments based on time dependence cannot explain the very high estimates for r/m for the South American sub-group, as this sub-group emerged more recently than the Turkish and Asian sub-groups. An alternative explanation to time-dependent effects is variation in transmission patterns within and between each geographic region, owing to host movement rather than intrinsic differences in transmissibility of the bacteria. Harris *et al. *[2] noted evidence for an inter-continental transmission event from South America to Portugal, which is likely to have initiated the wave of ST239 cases in this country (the 'Brazilian clone') in the late 1990s/early 2000s (the green cluster within the South American group in Figure 3). It is possible that this transmission event increased the opportunity for recombination with European ST239 lineages, notably the 'Portuguese clone' of ST239, which caused the prior wave of ST239 infection in Portugal in the early 1990s [33]. However, the admixture analysis does not help to explain the high rates of recombination in the South American sub-group, as this analysis suggests a higher rate of gene flow between Europe and Asia. In contrast, our data suggest both very low rates of transmission of ST239 into or out of Turkey, and low admixture between the Turkish sub-group and other groups. This relative isolation helps to explain why the r/m for the Turkish group is lower than for the Asian sub-group, despite these groups having emerged at approximately the same time. An important caveat is that the Asian sub-group (which includes a small number of isolates from China) will be substantially larger than the Turkish sub-group in terms of potential hosts, and that the definition of the sub-groups depends on the sampling frame, as discussed above.

Finally, it is possible that genomic changes such as the acquisition of a virulence factor may result in an increase in transmissibility and an epidemiological shift that, in turn, impacts on local rates of recombination. The acquisition of sasX by ST239 in Eastern China [11] is a notable example. However, there is no clear evidence for broad epidemiological distinctions between the sub-groups compared in this analysis. It is also possible that genetic differences, such as the presence of a particular phage variant or restriction modification system, might impact on the intrinsic rate of recombination.

## Conclusions

We have shown variation in rates of recombination within a robust phylogeographic framework, and speculated as to the likely forces underpinning these differences. A great deal more theoretical and empirical work is required to fully understand this variation, in particular the respective roles of transmission and the chances that two different lineages will meet, time-dependence and the role of purifying selection, and genomic changes impacting on recombination rate. Although the forces responsible for intra-clonal and intra-species variation in recombination rates remain unclear, here we demonstrate the utility of a closer synthesis of phylogeography and molecular dynamics.

## Materials and methods

### The data set and genome sequencing

We supplemented a previous MRSA ST239 data set [2] consisting of isolates taken from global sources (*n *= 42) and a single hospital in Thailand (*n *= 20) with 71 clinical isolates from four Turkish hospitals. These isolates were all sampled between 2006 and 2009 and represent a range of sources (for example, blood, catheter, swab; details are provided in Additional file 13); 31 clinical isolates from a global collection were also sequenced, giving a total of 165 isolates for this study. ST239 is a heath-care associated (HA) MRSA lineage and all the isolates used in this study were collected from hospitals. A list of all the strains is provided in Additional file 1. Unique index-tagged libraries for each sample were created, and up to 12 separate libraries were sequenced in each of eight channels in Illumina Genome Analyser GAII cells with 75-base paired-end reads. Data have been deposited in the European Nucleotide Archive under study number ERP000228. The paired-end reads were mapped against the chromosome of *S. aureus *TW20 (accession number FN433596) [13] using SMALT [34] and SNPs were identified as described in [1]. Mobile genetic elements and accessory regions in the TW20 reference chromosome had previously been identified by manual curation [13]. Coding sequences in TW20 were assigned to functional role categories adapted from Riley [19] by manual curation [13].

### Tests for recombination

We applied the BRATNextGen software on the whole genome alignment with the same default settings as in [17]. Significance of each putative recombinant segment (*P*-value <0.05) was determined through a bootstrap test with 100 replicate analyses executed in parallel on separate processors. As shown by Marttinen *et al. *[17], this approach yielded almost identical results with the analysis of the PMEN1 data in Croucher *et al. *[1], but was considerably faster and also directly indicates which recombination events are shared by subsets of isolates. Neighbor net and the PhiTtest were implemented using SplitTree4 [18].

### Maximum likelihood phylogeny

A maximum likelihood phylogenetic analysis was run using PhyML [35]. We used a multiple alignment composed of all the SNPs that have not been affected by recombination. As we were only considering SNPs, the proportion of invariable sites was set to 0. The General Time-Reversible (GTR) model was used, where the base frequencies and the relative substitution rates between them were estimated by maximizing the likelihood of the phylogeny. For estimating the tree topology both nearest neighbor interchange and subtree pruning and regrafting methods were used. To measure the group support across the phylogeny, 100 non-parametric bootstrap replicates were run.

### Bayesian Analysis of Population Structure

We conducted the mixture and admixture analyses using BAPS version 5.3 [20], converting the SNP alignment file into the appropriate format using a PERL script. The 'Clustering of individuals' module was used for the population mixture analysis. In order to evaluate the consistency of the results a vector from 2 to 20 K values (where K is the maximum number of groups) was run. For the population admixture analysis, the 'Admixture based on mixture clustering' was run with a minimum population size of 5 and the following specifications: the iterations to estimate the admixture coefficient for the individuals were 100; the reference individuals from each population were 200; and the number of iterations for estimating the admixture coefficient for reference individuals was 20. The *P*-values in Table 1 were computed by BAPS during the admixture analysis as described in the BAPS manual [36] (p. 25): 'The final column gives the *p*-value for the individual. This value tells the proportion of reference individuals simulated from the population in which the individual was originally clustered having the admixture coefficient to the cluster smaller than or equal to the individual.'

### Bayesian Markov Chain Monte Carlo analyses

The dated phylogeny analysis was implemented through BEAST [21]. For this analysis a multiple alignment of all the SNPs not affected by recombination was used. Since we had the date of sampling for the isolates, we could use this information for dating. We used the uncorrelated log-normal relaxed clock with the GTR model and did not use the correction for site rate heterogeneity because the statistical model selection did not show this as a significant parameter. The analysis was run for 90,000,000 steps and at every 9,000 steps samples were taken. We discarded 9,000,000 steps as burn-in. The program TRACER was used to evaluate the convergence of the analysis. This was done by looking at the trace plots of the likelihood scores and by confirming that the effective sample size of the tree likelihood was greater than 200.

### Statistical analyses and r/m ratios

All the statistical analyses were carried out via R [37]. The r/m ratios were calculated from the output of BRATNextGen using a PERL script.

## Abbreviations

BAPS: Bayesian Analysis of Population Structure; BEAST: Bayesian Evolutionary Analysis by Sampling Trees; GTR: General Time-Reversible; MGE: mobile genetic element; MLST: multilocus sequence typing; MRSA: methicillin-resistant *Staphylococcus aureus*; NGS: next-generation sequencing; SNP: single-nucleotide polymorphism.

## Authors' contributions

SCR conducted most of the analyses. JC and PM ran the BRATNextGen analysis. MTH carried out the genome sequencing and ran the method for detecting recombination used in [1]. WPH, HW, KB, ZG, MA, SDB and JP have made substantial contributions to acquisition and interpretation of data. EJF coordinated the study. SCR and EJF designed the study, analyzed the results, and wrote the manuscript. JC, WPH, and HW critically revised the manuscript. All authors carefully read and approved the final manuscript.

## Acknowledgements

This work was funded by the TROCAR consortium (EU FP7-HEALTH #223031) and the UK Clinical Research Collaboration Translational Infection Research Initiative (grant number G1000803). We thank the core sequencing and informatics teams at the Sanger Institute for their assistance and The Wellcome Trust for its support of the Sanger Institute Pathogen Genomics and Biology groups. MTGH, SDB and JP were supported by Wellcome Trust grant 098051. We are truly thankful to the reviewers whose comments have significantly improved our manuscript.

## Supplementary Material

Additional file 1 **List of the strains used in this study**. This file lists all the strains used as well as their country of origin. The second column states whether the strain was sequenced for this study (newly sequenced) or the strain came from the study by Harris *et al. *[2] (previously sequenced). Click here for file

Additional file 2 **Recombination events detected via BRATNextGen**. Details of the recombination events, for every strain the recombination events are listed as well as the boundaries of these events. Click here for file

Additional file 3 **Neighbor net based on all the SNPs**. Tip labels were taken out for the sake of clarity. The network was constructed by means of SplitTree and shows extensive reticulation consistent with the conflicting phylogenetic signals created by recombination. Additionally, we used the Phi test also through SplitTree to further corroborate the presence of recombination. The test was statistically significant with a *P*-value equal to 0. Click here for file

Additional file 4 **List of the genes affected by recombination based only on BRATNexGen results**. Genes were considered recombinant if more than 100 bp of their sequence have undergone recombination based on the BRATNexGen output. The gene identifier is with respect to the reference genome TW20. The total number of genes affected by recombination according to this criterion was 441. Click here for file

Additional file 5 **Table showing MGEs used for the r/m analyses**. The description and coordinates of the MGEs are with respect to the reference genome TW20. Click here for file

Additional file 6 **List of the genes affected by recombination based on BRATNexGen and the method used in**[1]. Genes were considered recombinant if more than 100 bp of their sequence have undergone recombination based on the BRATNexGen output and the results from the method used in [1]. The gene identifier is with respect to the reference genome TW20. The number of genes affected by recombination according to this criterion was 312. Click here for file

Additional file 7 **Functions affected by recombination using BRATNextGen and the method used in**[1]. The bars are the most inclusive categories based on an adapted version of Riley's classification. The blue section of the bars represents the percentage of genes of each functional category affected by recombination. The 'extrachromosomal' category was here renamed 'MGE' to avoid confusion as these genes are physically located on the chromosome. Click here for file

Additional file 8 **Multiple alignment used for constructing the phylogeny in Figure 3**. Multiple alignment in phylip format containing all the SNPs not affected by recombination that was used to construct the phylogeny shown in Figure 3 and to conduct the power calculation mentioned in the Discussion. Click here for file

Additional file 9 **Groups identified by BAPS**. For each group the list of the isolates composing it is given as well as the main geographic location of the group. Click here for file

Additional file 10 **Bayesian dated phylogeny constructed via BEAST**. For the sake of clarity the Turkish (dark blue triangle), South American (light blue triangle), and Asian (red triangle) groups were collapsed. The violet horizontal bars show the 95% highest posterior density (HPD) intervals for the divergence time estimates. Click here for file

Additional file 11 **Description of the populations used for the analysis of the founder events**. Three groups were defined and this table gives the list of isolates for each group and the main geographic region. Click here for file

Additional file 12 **Domestic flights between the three Turkish cities**. Total number of domestic flights between the three Turkish cities during the week 2 to 8 November 2012. Click here for file

Additional file 13 **Some clinical details of the newly sequenced Turkish isolates**. This file shows the isolation date and the source for most of the newly sequenced Turkish isolates. Click here for file
