# Supplementary figures and images for: Phylogeographic variation in recombination rates within a global clone of methicillin-resistant Staphylococcus aureus
Source: Genome Biol. 2012 Dec 27;13(12):R126. doi: 10.1186/gb-2012-13-12-r126 (PMC3803117; doi:10.1186/gb-2012-13-12-r126)

0.01

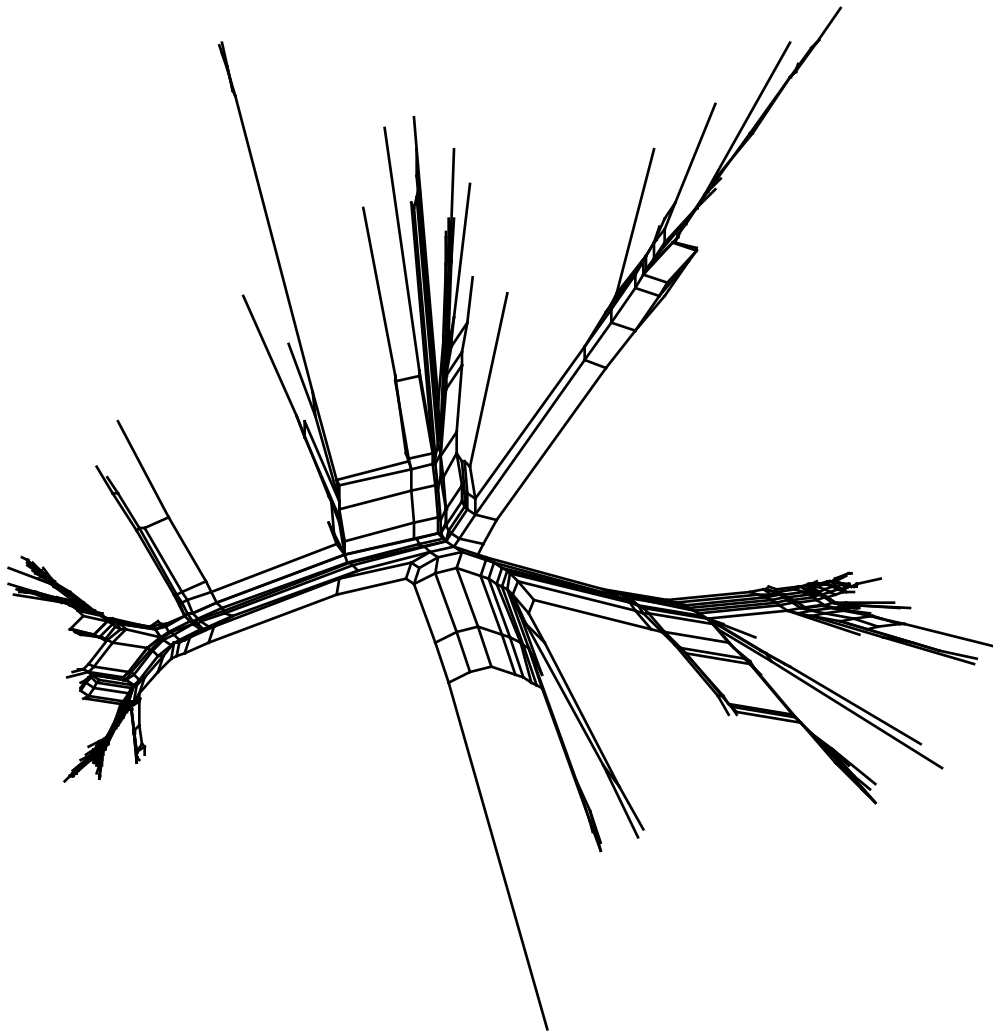

Supplement: Additional file 3 — Neighbor net based on all the SNPs. Tip labels were taken out for the sake of clarity. The network was constructed by means of SplitTree and shows extensive reticulation consistent with the conflicting phylogenetic signals created by recombination. Additionally, we used the Phi test also through SplitTree to further corroborate the presence of recombination. The test was statistically significant with a P-value equal to 0. [file gb-2012-13-12-r126-S3.PDF]

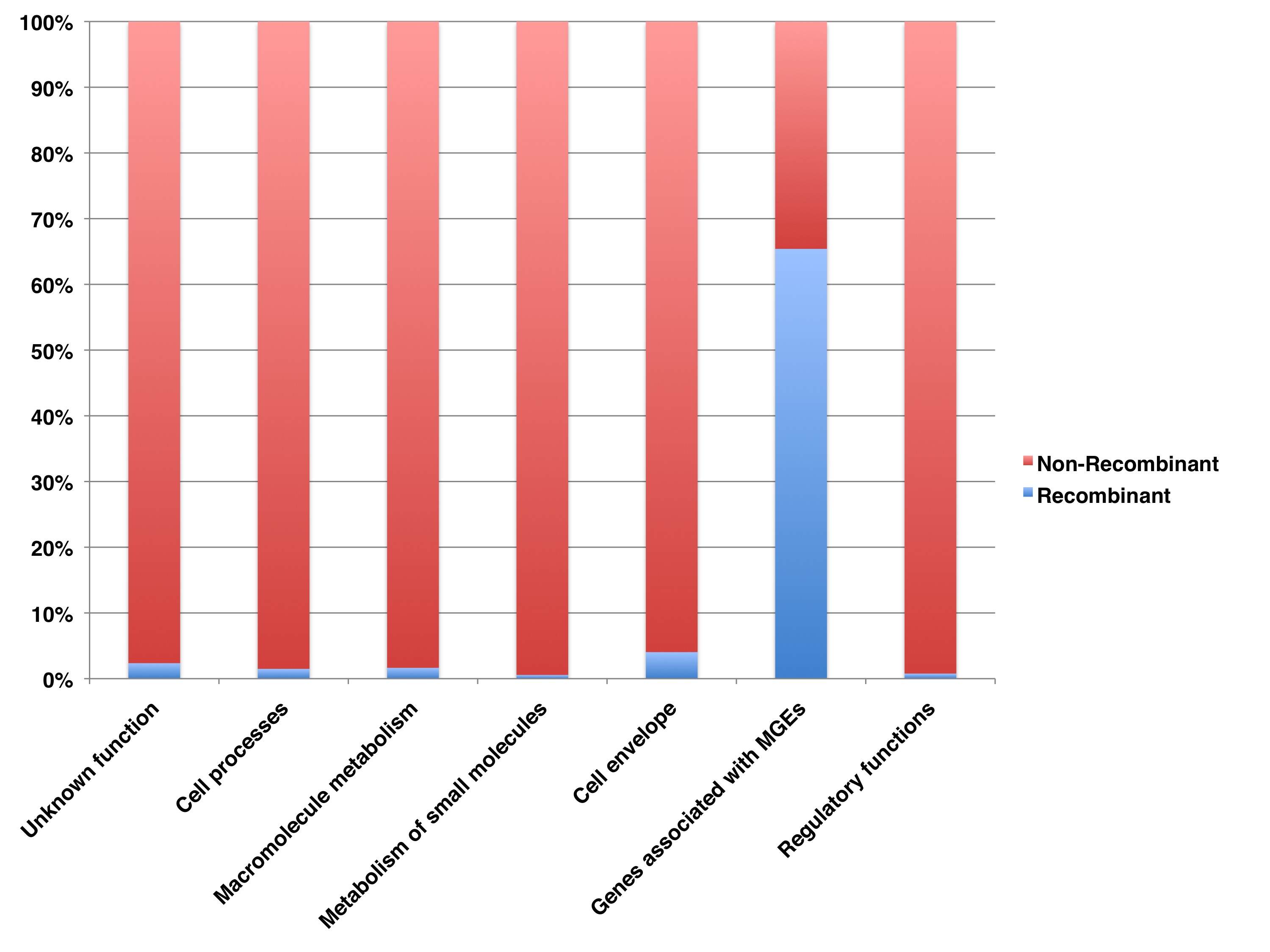

Supplement: Additional file 7 — Functions affected by recombination using BRATNextGen and the method used in[1]. The bars are the most inclusive categories based on an adapted version of Riley's classification. The blue section of the bars represents the percentage of genes of each functional category affected by recombination. The 'extrachromosomal' category was here renamed 'MGE' to avoid confusion as these genes are physically located on the chromosome. [file gb-2012-13-12-r126-S7.JPEG]

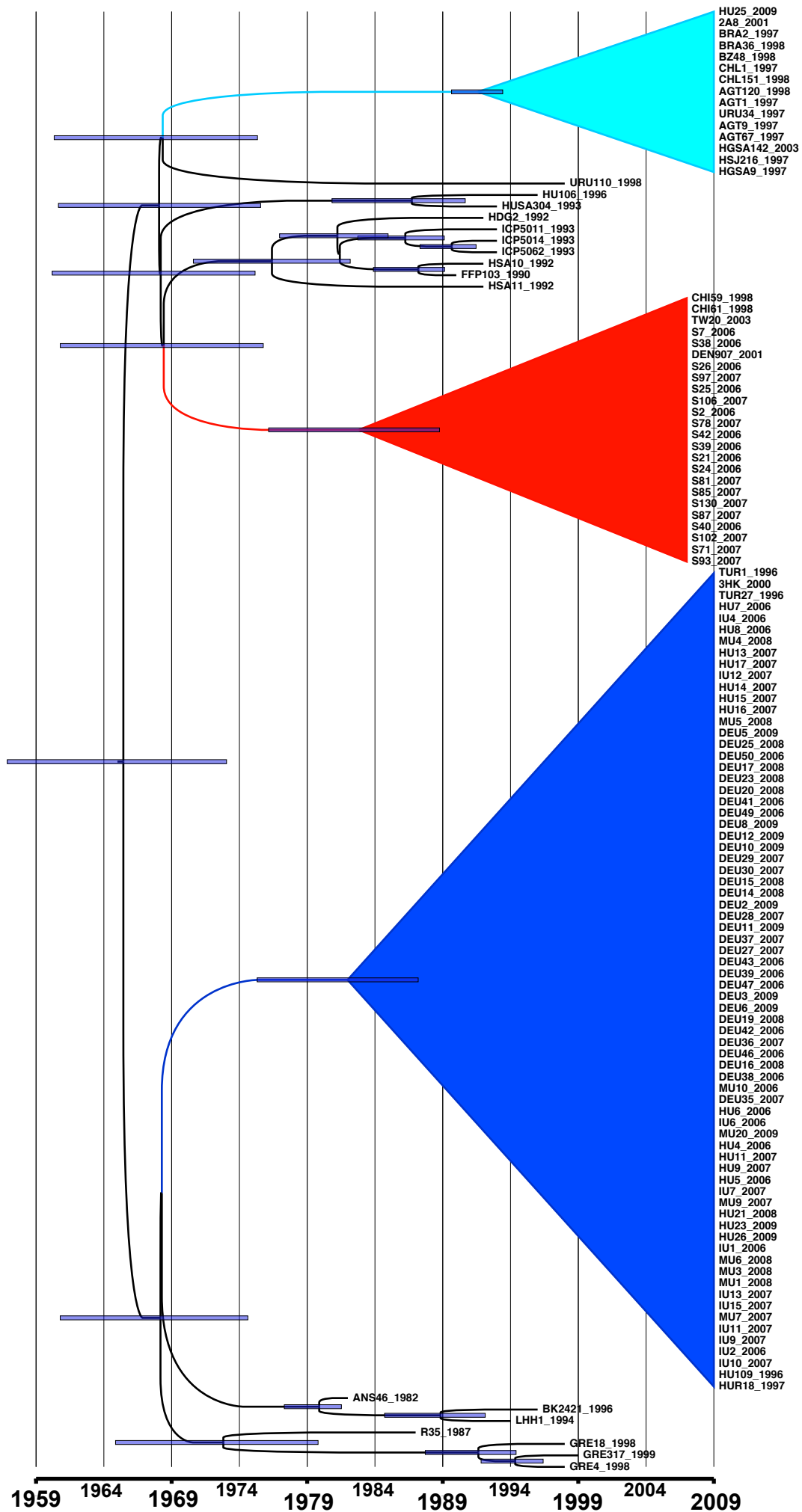

Supplement: Additional file 10 — Bayesian dated phylogeny constructed via BEAST. For the sake of clarity the Turkish (dark blue triangle), South American (light blue triangle), and Asian (red triangle) groups were collapsed. The violet horizontal bars show the 95% highest posterior density (HPD) intervals for the divergence time estimates. [file gb-2012-13-12-r126-S10.PDF]
