# Supplementary material for: Phylogeographic variation in recombination rates within a global clone of methicillin-resistant Staphylococcus aureus
Source: Genome Biol. 2012 Dec 27;13(12):R126. doi: 10.1186/gb-2012-13-12-r126 (PMC3803117; doi:10.1186/gb-2012-13-12-r126)
Supplement: Additional file 5 — Table showing MGEs used for the r/m analyses. The description and coordinates of the MGEs are with respect to the reference genome TW20. [file gb-2012-13-12-r126-S5.DOC]

**Table 5**

Description and coordinates of the MGE in the reference genome TW20.

| **MGE** | **Coordinates** |
| --- | --- |
| SCCmercury | 34122..67172 |
| SCCmec type III | 67173..102467 |
| Prophage phiSa1 | 376406..419745 |
| Putative transposon/ICE Tn5801 | 486820..518088 |
| Putative pathogenicity island | 952129..966671 |
| Transposon Tn554 | 1775192..1781878 |
| Prophage phiSa3 | 2108459..2153126 |
| Prophage phiSa5 | 2181686..2308888 |
| Integrative conjugative element ICE6013 | 2873696..2893550 |
