# Supplementary material for: Phylogeographic variation in recombination rates within a global clone of methicillin-resistant Staphylococcus aureus
Source: Genome Biol. 2012 Dec 27;13(12):R126. doi: 10.1186/gb-2012-13-12-r126 (PMC3803117; doi:10.1186/gb-2012-13-12-r126)
Supplement: Additional file 9 — Groups identified by BAPS. For each group the list of the isolates composing it is given as well as the main geographic location of the group. [file gb-2012-13-12-r126-S9.DOC]

Supplementary Table

Groups identified by BAPS

| Group | Main geographic region | List of isolates |
| --- | --- | --- |
| 1 | Turkey* | HU13 HU26 HU14 HU15 HU16 HU17 HU21 HU23 IU1 IU11 IU12 IU13 IU2 IU4 IU6 IU7 IU9 IU10 IU15 IU17 IU18 IU19 IU20 H482 MU3 MU4 MU5 MU6 MU7 MU9 MU10 DEU10 DEU11 DEU12 DEU2 DEU3 DEU5 DEU6 DEU8 DEU23 DEU25 DEU14 DEU15 DEU16 DEU17 DEU19 DEU20 DEU37 DEU38 DEU39 DEU27 DEU28 DEU29 DEU30 DEU35 DEU36 DEU50 MU1 DEU41 DEU42 DEU43 DEU46 DEU47 DEU49 HU11 MU20 HU4 HU5 HU6 HU7 HU8 HU9 3HK TUR27 HU109 HUR18 TUR1 |
| 2 | Asia* | TW20 M705 MAL119 MAL215 MAL3 MAL9 H211 D71 MAL1 MAL11 M116 M1229 M170 M592 M996 UK102 D90 H202 H216 CHI59 CHI61 S85 S130 S87 S93 S71 S40 S102 S7 S38 DEN907 S26 S97 S25 S106 S2 S78 S42 S81 S24 S21 S39 |
| 3 | South America* | RA7 FRICAR ES26 M278 RA3 RA6 2A8 HU25 BRA2 CHL1 CHL151 BRA36 BZ48 AGT120 HSJ216 HGSA9 HGSA142 AGT9 AGT67 URU34 AGT1 |
| 4 | Europe+USA | NA32 H24 Na21 M418 P32 LIT89 LIT68 LIT76 ANS46 URU110 BK2421 LHH1 HU106 HUSA304 HSA11 HDG2 HSA10 FFP103 ICP5011 ICP5014 ICP5062 R35 GRE18 GRE317 GRE4 |

*These three groups are monophyletic in the Maximum Likelihood phylogeny
