# Supplementary material for: Phylogeographic variation in recombination rates within a global clone of methicillin-resistant Staphylococcus aureus
Source: Genome Biol. 2012 Dec 27;13(12):R126. doi: 10.1186/gb-2012-13-12-r126 (PMC3803117; doi:10.1186/gb-2012-13-12-r126)
Supplement: Additional file 11 — Description of the populations used for the analysis of the founder events. Three groups were defined and this table gives the list of isolates for each group and the main geographic region. [file gb-2012-13-12-r126-S11.DOC]

Supplementary Table

**Groups considered**

| Geographic region | List of isolates |
| --- | --- |
| Europe | GRE18 GRE4 GRE317 HU106 HUSA304 HSA11 HDG2 ICP5011 ICP5014 ICP5062 FFP103 HSA10 |
| Istanbul and Ankara | MU10 DEU35 MU7 IU15 IU13 HU6 IU6 MU20 HU23 HU21 MU9 IU7 HU4 HU5 HU11 HU9 IU18 HU26 MU3 MU1 IU1 MU6 IU19 HU15 HU14 MU5 HU16 IU9 IU11 IU2 IU10 IU17 IU20 HU17 HU13 IU12 MU4 HU8 IU4 HU7 |
| Izmir | DEU38 DEU46 DEU16 DEU36 DEU25 DEU5 DEU47 DEU19 DEU42 DEU3 DEU6 DEU12 DEU10 DEU8 DEU50 DEU17 DEU23 DEU20 DEU41 DEU49 DEU39 DEU30 DEU14 DEU27 DEU37 DEU28 DEU11 DEU15 DEU2 DEU29 DEU43 |
